# Hydrogen bond guided synthesis of close-packed one-dimensional graphdiyne on the Ag(111) surface†
†Electronic supplementary information (ESI) available. See DOI: 10.1039/c9sc04530a


**DOI:** 10.1039/c9sc04530a

**Published:** 2019-10-28

**Authors:** Zhi Chen, Tao Lin, Haohan Li, Fang Cheng, Chenliang Su, Kian Ping Loh

**Affiliations:** a SZU-NUS Collaborative Innovation Center for Optoelectronic Science & Technology , International Collaborative Laboratory of 2D Materials for Optoelectronics Science and Technology of Ministry of Education , Institute of Microscale Optoelectronics , Shenzhen University , Shenzhen , 518060 , China . Email: chmsuc@szu.edu.cn; b Department of Chemistry , Centre for Advanced 2D Materials (CA2DM) , National University of Singapore , 3 Science Drive 3 , Singapore 117543 , Singapore . Email: chmlohkp@nus.edu.sg; c College of New Materials and New Energies , Shenzhen Technology University , Shenzhen 518118 , China

## Abstract

Aided by hydrogen bonding, alkyne and oxazole functionalized precursors undergo uniform self-assembly, which serves as a template for the fabrication of one-dimensional graphdiyne-like wires on the Ag(111) surface.

## Abstract 2

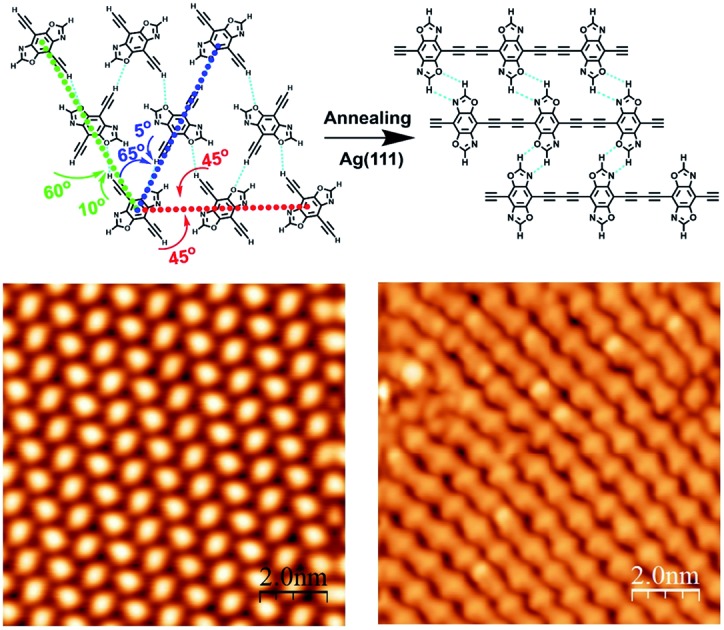
Aided by hydrogen bonding, alkyne and oxazole functionalized precursors undergo uniform self-assembly, which serves as a template for the fabrication of one-dimensional graphdiyne-like wires on the Ag(111) surface.

## Introduction

Recently, surface confined molecular engineering has attracted much attention, because it can be used to build one-dimensional (1D) chains or two-dimensional (2D) networks showing intriguing chemical and physical properties, with potential application in electronic devices, catalysis and separation.^1^ Molecules with terminal alkyne functional groups have been used as precursors for surface homo-coupling and cyclotrimerization to fabricate 1D and 2D structures.1a,d,g In 2010, the first artificial graphdiyne (GDY) was synthesized through homo-coupling of terminal alkynes on Cu foil.^2^ With directional anisotropy and tunable bandgap through versatile structural design, graphdiyne is a remarkable material beyond graphene.1a,d,g,^3^ However, the synthesis of good quality graphdiyne is still challenging and this limits the widespread application of this material. On-surface synthesis has been considered as a promising method to grow graphdiyne,^4^ although in practice the synthesis is challenged by side reactions and reduced reactivity after polymerization.

To overcome these obstacles, various strategies have been proposed to achieve highly ordered polymerization of molecules. One strategy is the use of a surface template. For example, the vicinal Ag(877) surface can steer the alignment of terminal alkyne-functionalized molecules along the step-edges of substrates. This suppresses the unwanted branching side reactions of terminal alkyne groups, facilitating the growth of 1D graphdiyne-related chains.4*c* Another strategy is the use of sterically hindered groups. For instance, alkane,4*b* benzoic ethynyl groups4*d* and polar carbonitrile groups4*g* have been introduced into alkyne derivatives to form 1D graphdiyne chains. However, the 1D chains synthesized by both strategies are sparsely distributed and lacked long range order.

It is well known that coordination bonds^5^ and hydrogen bonds^6^ can be used to assist the self-assembly of organic molecules to form large domains with low defect densities on the metal surface, on account of the reversibility of bond formations. In contrast, the irreversibility of the covalent bond formation prevents self-repair, thus it is challenging to form highly periodic networks using covalent bonds alone. Instead, coordination or hydrogen bonds are a better choice for pre-packing the monomer into a highly crystalline network, which can serve subsequently as a template for intermolecular covalent cross-linking to form 1D or 2D conjugated polymers. In such a pre-packed assembly, the organic monomer only needs to move a short distance or rotate a small angle to trigger the coupling reaction under thermal annealing or UV radiation. A metal-free assisted assembly affords unique advantages; as there will be no residual metal atoms after the coupling reaction, the latter can cause a structural disorder and also affect device performance.^7^

**Fig. 1:**
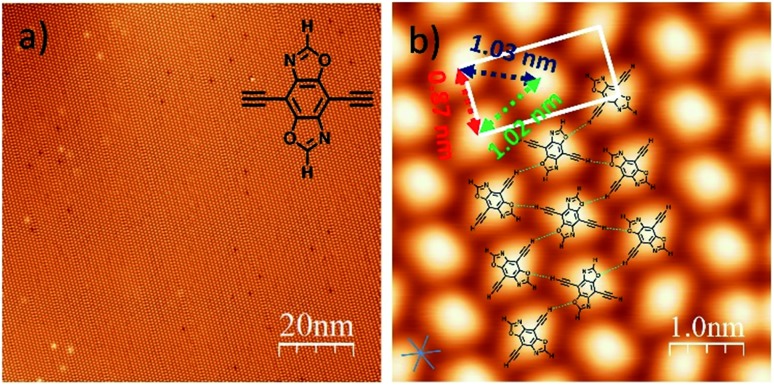
(a) STM image of the self-assembly of **DEBBA** on a Ag(111) surface. Inset: a chemical structure of **DEBBA**. (b) High-resolution STM image of the self-assembled structure of **DEBBA** on Ag(111). The directions of the close-packed substrate are indicated at the lower left with a blue star. The close-packed **DEBBA** directions are indicated with a red-green-blue dashed triangle, which is along the directions are indicated with a red-green-blue dashed triangle, which is along the 〈112̄〉 directions of Ag(111). Scanning parameters for (a and b): 112[combining macron] directions are indicated with a red-green-blue dashed triangle, which is along the 〈112̄〉 directions of Ag(111). Scanning parameters for (a and b): directions of Ag(111). Scanning parameters for (a and b): *U* = –1.5 V; *I* = 0.1 nA.

Here, we introduced a hydrogen bond donor and acceptor group oxazole onto the alkyne derivative backbones and synthesized the precursor 4,8-diethynylbenzo[1,2-*d*-4,5-*d*′]bisoxazole (**DEBBA**) (Fig. 1a, inset), with the objective of improving the chemoselectivity of homo-coupling of alkynes. In **DEBBA**, the oxazole group has an O atom and N atom as hydrogen acceptors and also a relatively active C–H as a hydrogen donor at the middle position between O and N atoms. These functionalities impart on **DEBBA** the ability to participate in network formation.

## Results and discussion

The precursor **DEBBA** was synthesized by following a reported procedure.^8^ All scanning tunneling microscopy (STM) experiments were performed using a commercial ultrahigh vacuum (UHV) scanning tunneling microscope. A single-crystalline Ag(111) substrate was cleaned by repeated cycles of Ar^+^ sputtering and annealing at about 500 °C. **DEBBA** molecules were then evaporated from a quartz crucible heated at 120 °C onto the clean Ag(111) substrate held at room temperature (RT). The sample was subsequently annealed at different temperatures. The STM tip used here was a commercial Pt–Ir wire. All STM images were acquired at –196 °C, in the constant current mode and processed by WSxM.^9^

After the deposition of **DEBBA** onto Ag(111) at room temperature, a large area self-assembled network of **DEBBA** was formed (Fig. 1a). The high-resolution STM image (Fig. 1b) indicates that an individual molecule of **DEBBA** shows ellipsoid-like protrusions and follows a chevron-type arrangement with a rectangular lattice defined by unit cell lengths of *a* = 0.87(2) nm and *b* = 1.82(3) nm. The close-packed **DEBBA** directions are indicated with a red-green-blue dashed triangle, which is along the directions are indicated with a red-green-blue dashed triangle, which is along the 〈112̄〉 directions of Ag(111). The three kinds of distance between two nearby 112[combining macron] directions are indicated with a red-green-blue dashed triangle, which is along the 〈112̄〉 directions of Ag(111). The three kinds of distance between two nearby directions of Ag(111). The three kinds of distance between two nearby **DEBBA** molecules are 1.03(2) nm, 1.02(2) nm and 0.87(2) nm, respectively (Fig. 1b). The superimposed structural model shows that the monolayer pattern is mainly formed by C–H···O hydrogen-bonding between adjacent ligands. The C–H in the terminal alkyne group plays the role of a proton donor and the O atom in the oxazole group acts as a proton acceptor. As a result, each **DEBBA** molecule forms four hydrogen-bonds with the neighbouring four **DEBBA** molecules. Due to the hydrogen bond interaction, the self-assembled network is highly uniform and has few defects.

**Fig. 2:**
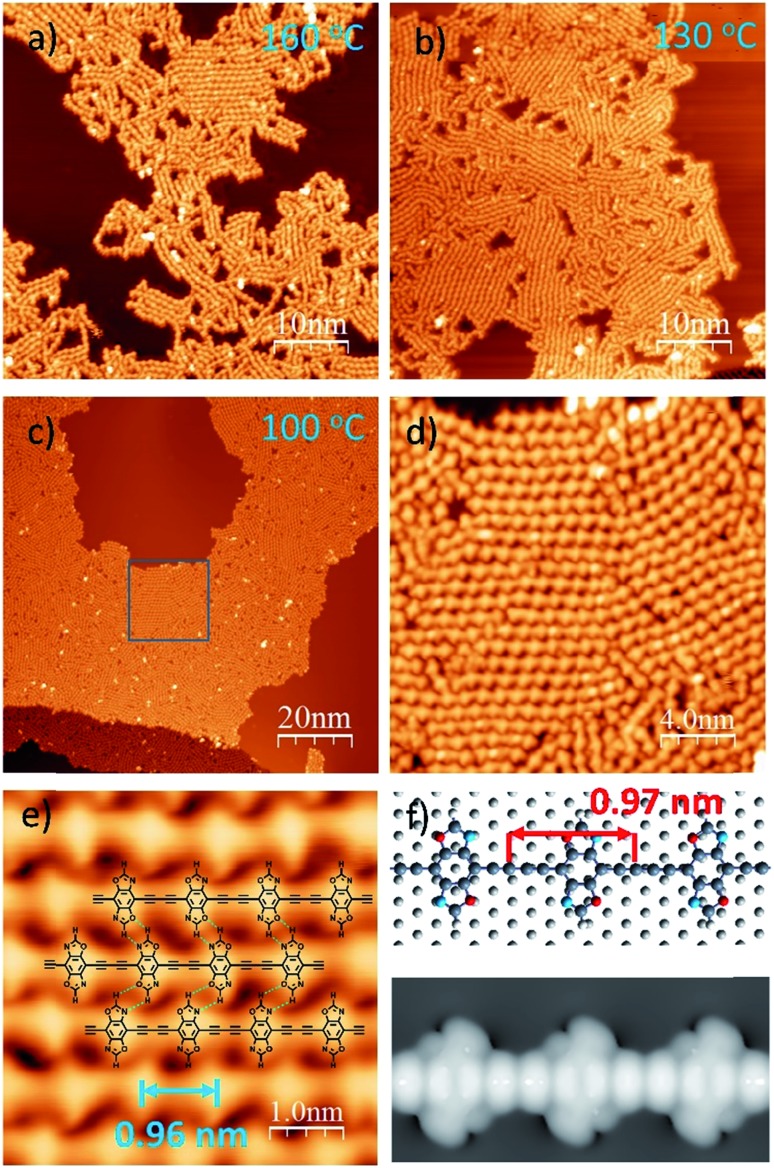
STM images of 1D graphdiyne formed by annealing the self-assembled **DEBBA** network at different temperatures: 160 °C, 10 min (a); 130 °C 30 min (b); 100 °C 1 hour (c–e). Scanning parameters for (a–e): *U* = –1.5 V; *I* = 0.1 nA. (f) The energetically favored adsorption geometry and STM image (*U* = –1.5 V) of 1D graphdiyne on Ag (111), obtained from DFT calculations.

To induce C–C covalent coupling between the pre-packed molecular network, we chose different annealing temperatures. After the sample was annealed at 160 °C for 10 min (Fig. 2a), 1D linear chain segments were observed on the Ag(111) substrate, indicating that thermal treatment is able to trigger alkyne homo-coupling to form graphdiyne wires. However, the majority of the wires are randomly distributed on Ag(111). Annealing at a lower temperature of 130 °C allows the self-assembly of **DEBBA** to form multiple domains (Fig. 2b), where each domain consists of ordered arrangement of 1D graphdiyne chains. However, vacancies are present in domains and the assembly is not closely packed.

To further check how annealing temperature and time affect the packing of 1D graphdiyne, a one-hour annealing at 100 °C was performed. As shown in Fig. 2c–e, and S2,† highly ordered, closely packed 1D graphdiyne with an area larger than 100 × 50 nm^2^ can be observed on the Ag(111) surface. Each domain consists of parallelly aligned 1D graphdiyne wires. The distance of two oxazole repeat units is 0.96(2) nm, which is in good agreement with the length of 0.97 nm obtained from the Density Functional Theory (DFT) calculated model on the Ag(111) surface (Fig. 2f). The optimized model of graphdiyne has an almost flat lying molecular geometry on Ag(111) (Fig. 2f). The corresponding simulated STM image obtained at a bias voltage of –1.5 V (Fig. 2f) is in good agreement with the experimental results (Fig. 2e). To analyse the electronic properties of 1D graphdiyne, we calculated the electronic band structure of an infinite defect-free 1D graphdiyne wire using DFT (Fig. S4†). The energy gap is about 1.2 eV and 1D graphdiyne is a direct bandgap organic semiconductor.

**Fig. 3:**
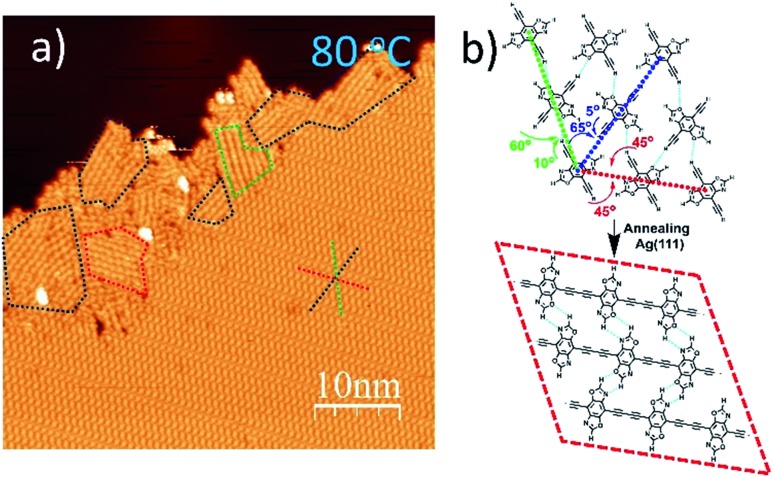
(a) STM image of self-assembled **DEBBA** and 1D graphdiyne products at the edge of self-assembly after 80 °C annealing for 30 min. The close-packed **DEBBA** directions are indicated with a red-green-blue dashed star. The red, green and blue dashed boxes show that the formation of 1D chains requires 45°, 60°, and 65° rotation of **DEBBA** molecules, respectively. Scanning parameters: *U* = –1.5 V; *I* = 0.1 nA. (b) Hydrogen bond-assisted self-assembly model of **DEBBA**, and its transformation into homo-coupled 1D graphdiyne wires.

When annealed at an even lower temperature of 80 °C, the polymerized products and the self-assembled **DEBBA** molecules coexist (Fig. 3a) which is different from fully homo-coupled ones at higher temperature annealing. Studying the image reveals the homo-coupling reactions started from the edge of the self-assembled domain and extended to the centre. The reacted domains (as shown in the red, green, and blue dashed boxes in Fig. 3a) mainly follow the three close-packed directions of **DEBBA** self-assembly (as red, green and dark blue dashed star in Fig. 3a). The red dashed box follows the repeating packing direction (red dashed line) of the self-assembly of **DEBBA**, and each molecule is required to rotate 45° to form the 1D graphdiyne chain (Fig. 3b up, red dashed line). The blue and green boxes follow the zigzag packing directions (blue and green dashed line) of the self-assembly, in which alternative molecules need to rotate 60° (green dashed line) or 65° (blue dashed line) to form the 1D graphdiyne chains (Fig. 3b). Because the rotation angles are not large, the occupied area by the **DEBBA** molecules only becomes 3.0% smaller after homo-coupling under a mild annealing condition; thus 1D graphdiyne chains remain closely packed. The close packing of graphdiyne wires is assisted by interchain hydrogen bonding.

Annealing at a high temperature of 250 °C is also performed to check whether any further reaction occurs in this system. As shown in Fig. S3,† the 1D wires become deformed due to thermally induced motion and interaction with the Ag substrate at high temperature. Hence, high temperature annealing is not beneficial for the ordered self-assembly of 1D graphdiyne.

## Conclusions

In conclusion, we have synthesized a precursor **DEBBA** that contains both alkyne and oxazole groups. These linkers allow the molecules to undergo self-assembly on the Ag(111) surface to form a pre-packed hydrogen-bonded network, which can be subsequently annealed to generate close-packed 1D graphdiyne wires. This work provides a new route to the synthesis of covalently linked 1D organic structures that are packed together by hydrogen bonds into a supramolecular framework.

## Conflicts of interest

There are no conflicts to declare.

## Supplementary Material

Supplementary information
