# Supplementary material for: Hydrogen bond guided synthesis of close-packed one-dimensional graphdiyne on the Ag(111) surface
Source: Chem Sci. 2019 Oct 28;10(47):10849–52. doi: 10.1039/c9sc04530a (PMC7003942; doi:10.1039/c9sc04530a)
Supplement: Supplementary file 1 [file SC-010-C9SC04530A-s001.pdf]

Electronic Supplementary Material (ESI) for Chemical Science.
This journal is © The Royal Society of Chemistry 2019

Supporting Information

Hydrogen Bond Guided Synthesis of Close-packed One-dimensional Graphdiyne on Ag(111) Surface

Zhi Chen, Tao Lin, Haohan Li, Fang Cheng, Chenliang Su,* and Kian Ping Loh*

Experimental

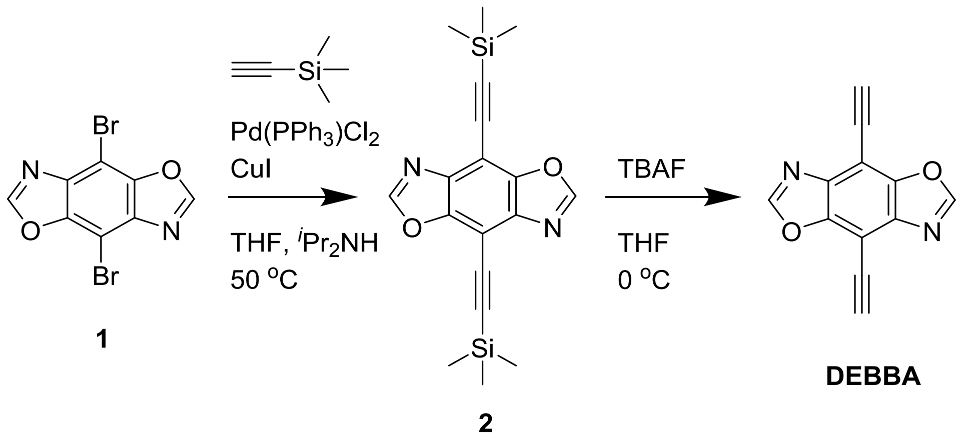

Scheme S1. The synthetic route of **DEBBA**.

4,8-Bis(trimethylsilylethynyl)benzo[1,2-*d*-4,5-*d′*]bisoxazole (2)

A dry three-neck, round-bottom flask equipped with a reflux condenser and addition funnel was placed under argon atmosphere and charged with 4,8-dibromobenzo[1,2-*d*-4,5-*d′*]bisoxazole (**1**) (2.510 g, 7.9 mmol) which was synthesized following literature,[1] $PdCl_2(PPh_3)_2$ (0.291 g, 0.4 mmol), CuI (0.160 g, 0.8 mmol), 50 mL dry/degassed diisopropylamine and 50 mL of dry/degassed THF. 2.8 mL trimethylsilylacetylene (1.960 g, 20 mmol) was added dropwise at room temperature and the mixture heated to 50 °C overnight. The mixture was allowed to cool to room temperature, and the volatile components were removed in vacuum. The crude product was purified by column chromatography on silica gel (hexane: CH_2Cl_2 = 2:1) affording white needle product **2** (1.264 g, 45% yield). ^{1}H NMR (500 MHz, $CDCl_3$) δ, ppm 8.25 (2H, s), 0.36 (18H, s);

^{13}C NMR (125 MHz, $CDCl_3$) δ, ppm 154.4, 148.5, 139.6, 108.2, 100.1, 93.4, 0.0; MS (EI) calcd for $C_{18}H_{20}N_2O_2Si_2$ 352.1 $[M]^+$, found 352.1.

4,8-Diethynylbenzo[1,2-*d*-4,5-*d′*]bisoxazole (DEBBA)

Compound **2** (0.176 g, 0.5 mmol) was dissolved in THF (20 mL) under an argon atmosphere at 0 °C. Then tetrabutylammonium fluoride (0.630 g, 2 mmol) was added. After 30 min, the reaction was poured into 100 mL deionized water. The precipitate was filtered and washed by methanol and hexane. The product was collected to yield a little yellow solid **DEBBA** (0.095 g, yield 92 %).

^{1}H NMR (500 MHz, $CDCl_3$) δ ppm 8.27 (2H, s), 3.86 (2H, s); ^{13}C NMR (125 MHz, D_6-DMSO) δ ppm 156.9, 148.3, 139.9, 98.6, 92.7, 73.9; MS (EI) calcd for $C_{12}H_4N_2O_2$ 208.0 $[M]^+$, found 208.0.

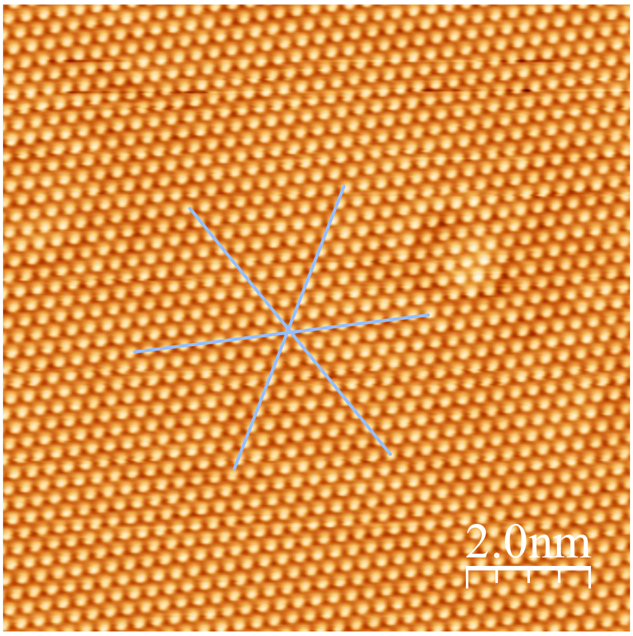

Figure S1. Atomic resolution STM image of Ag(111). The blue star shows the three directions of close-packing of Ag atoms. Scanning parameter: U = -0.26 V, I = 0.5 nA.

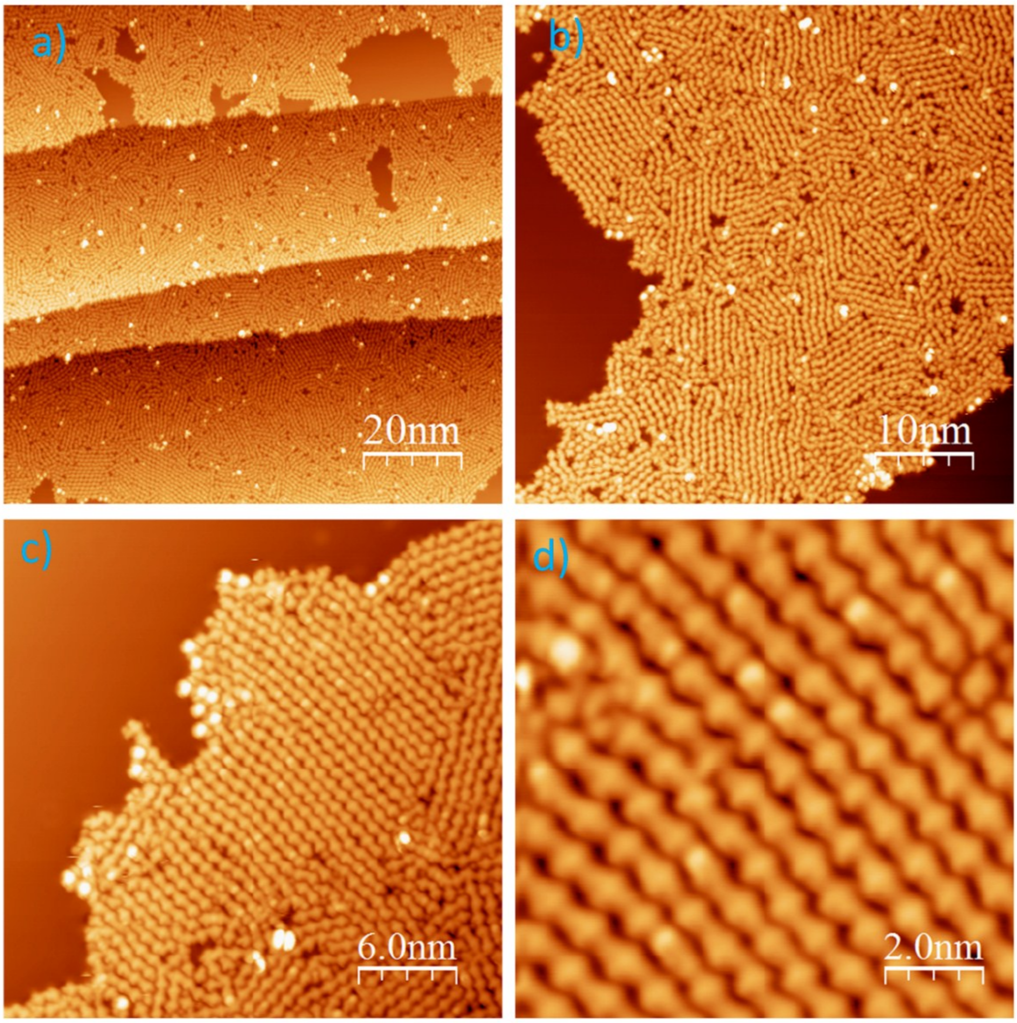

Figure S2. STM images of 1D Graphdiyne after annealing **DEBBA** self-assembly at 100 ^{o}C for 1 hour. Scanning parameter for (a-d): U = -1.5 V, I = 0.1 nA. At some edges of these self-assembled wire domains, there are some white contrast protrusions, which may originate from vertically oriented DEBBA molecules.

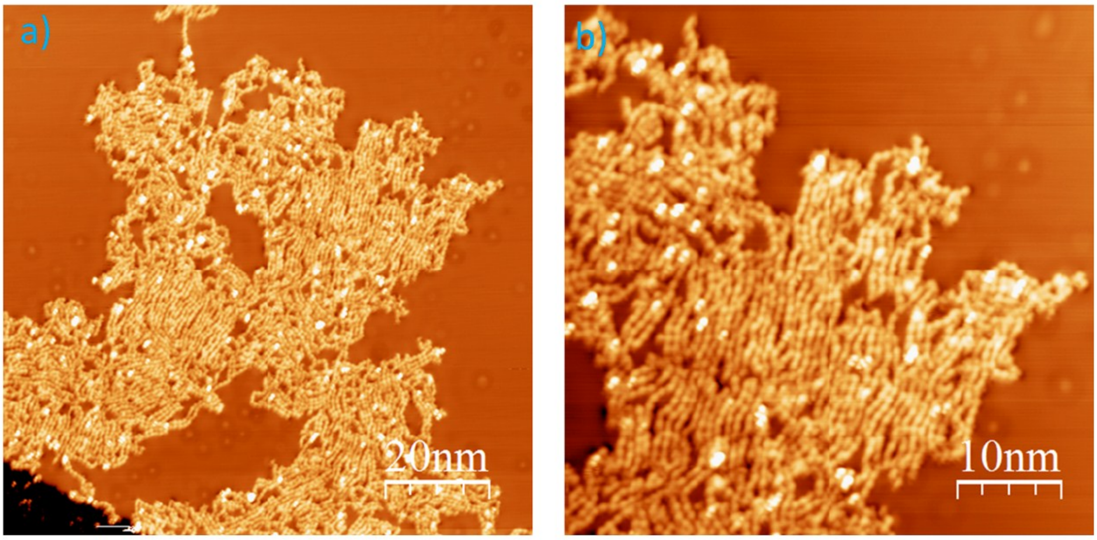

Figure S3. STM images of 1D graphdiyne which was formed by annealing **DEBBA**

self-assembly at 250 °C 10 min. Scanning parameter: U = -1.5 V, I = 0.1 nA.

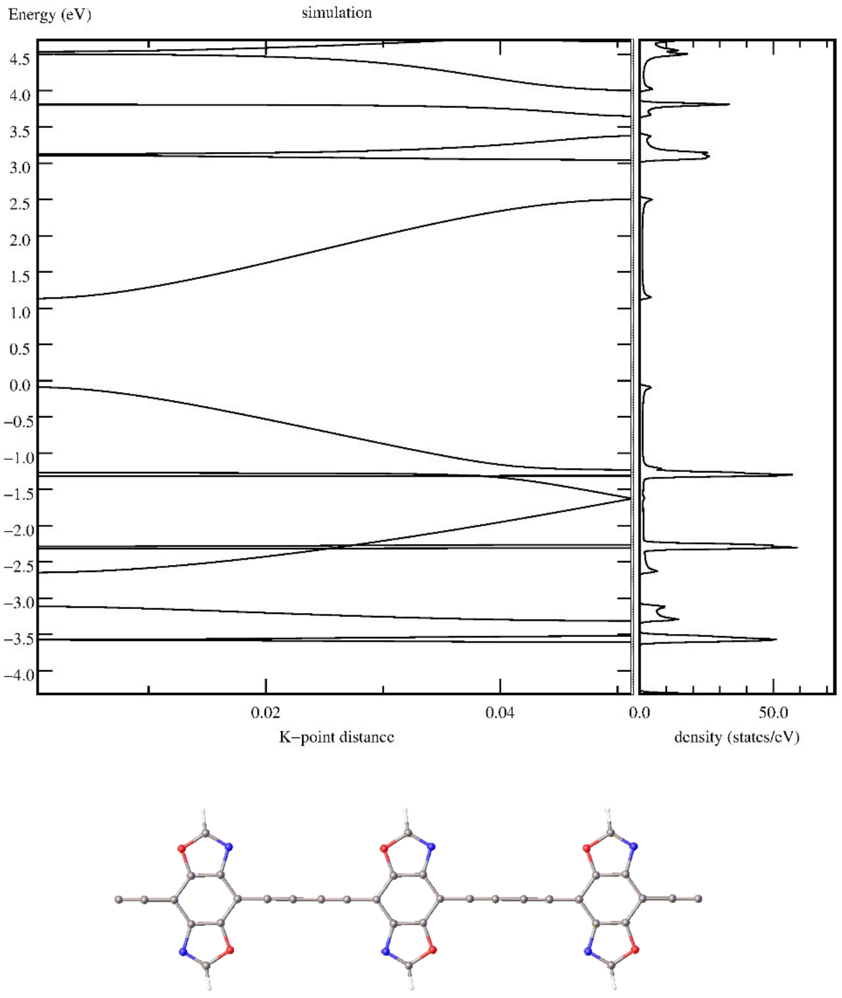

Figure S4. Electronic band structure and DOS (up) obtained from DFT calculations of an infinite 1D graphdiyne (down).

1 B. C. Tlach, A. L. Tomlinson, A. Bhuwalka and M. Jeffries-El, *J. Org. Chem.*, 2011, **76**, 8670.
